# Cord Blood Spexin Level in Mothers with Obesity—Forecast of Future Obesity?

**DOI:** 10.3390/children10091517

**Published:** 2023-09-06

**Authors:** Malgorzata Wojciechowska, Pawel A. Kolodziejski, Ewa Pruszynska-Oszmalek, Natalia Leciejewska, Hanna Krauss, Zuzanna Checinska-Maciejewska, Maciej Sassek, Anna Rekas-Dudziak, Malgorzata Bernatek, Marek Skrzypski, Maciej Wilczak

**Affiliations:** 1 Department of Mother and Child Health, Poznan University of Medical Sciences, 61-806 Poznan, Poland; malgorzata59@onet.eu (M.W.); maciej.sassek@up.poznan.pl (M.S.); kzmid@gpsk.am.poznan.pl (M.W.); 2 Department of Animal Physiology, Biochemistry and Biostructure, Faculty of Veterinary Medicine and Animal Science, Poznan University of Life Sciences, Wolynska Street 35, 60-637 Poznan, Poland; ewa.pruszynska@up.poznan.pl (E.P.-O.); natalia.leciejewska@up.poznan.pl (N.L.); marek.skrzypski@up.poznan.pl (M.S.); 3 Department of Medicine, The President Stanisław Wojciechowski State University of Applied Sciences in Kalisz, 62-800 Kalisz, Poland; hjk12@poczta.fm (H.K.); zuchecinska@gmail.com (Z.C.-M.); 4 Department of Anaesthesiology and Intensive Care, Hospital of the Ministry of the Internal Affairs and Administration, 60-631 Poznan, Poland; annarekasdudziak@gmail.com; 5 State Hospital Jarocin, Szpitalna 1, 63-200 Jarocin, Poland; drpiatek@o2.pl

**Keywords:** spexin, obesity, umbilical cord blood

## Abstract

Spexin (SPX) is a peptide that plays an important role in the regulation of food intake and body weight (BW) by the effect on carbohydrate-lipid metabolism. However, the role of SPX in fetal life, in children, and in adolescent metabolism is limited. Therefore, we decided to check whether obesity affects the concentration of SPX in the mother’s peripheral blood (MB) and umbilical cord blood (UCB). Using MB and UCB sera on the day of delivery obtained from 48 women (24 non-obese and 24 obese) and commercially available Elisa kits and colorimetric assays, we determined changes in SPX and the relationship between SPX concentration and other metabolic and anthropometric markers (body weight and BMI) on the day of delivery and in children at the age of 36 months. We found lower concentrations of SPX in MB (*p* < 0.05) and UCB (*p* < 0.01) derived from obese women (BMI > 30) and a moderate linear correlation (r = 0.4429; *p* < 0.01) between SPX concentrations in MB and UCB. We also noted that the concentration of SPX is not correlated with the child’s body weight on the day of birth (r = −0.0128). However, there is a relationship between SPX at birth and body weight at 3 years of age (r = −0.3219; *p* < 0.05). Based on the obtained results, it can be assumed that spexin is one of the factors modulating the child’s metabolism already in the fetal period and can be considered a potential marker of future predisposition to obesity. However, confirmation of this thesis requires additional research.

## 1. Introduction

Every year, bioinformatics research aimed at searching for previously unknown biologically active substances produced by the human body provides information on new peptides, neuropeptides, and proteins that perform important metabolic functions. One of these substances that has become known relatively recently is spexin. Spexin (SPX, also called neuropeptide Q (NPQ)) is a very conservative 14-amino acid peptide described for the first time in 2007 [1]. Still, little is known about the function of this peptide. So far, it has been shown that it is a strong regulator of carbohydrate-lipid metabolism and body weight, and the level of SPX appears to be altered in metabolic disorders [2,3,4,5]. It has also been shown that the level of SPX is lower in the serum and adipose tissue of obese, insulin-resistant, and/or diabetic patients and is closely correlated with body weight and other metabolic markers [6,7,8,9,10]. The latest studies using laboratory animals and in vitro models show that this peptide is very important in regulating the body’s lipid metabolism, and its deficiencies are correlated with higher body weight [11,12]. It has also been shown that the concentration of SPX can be modulated by exercise [13]. However, this is still a matter of debate due to discrepancies in the results obtained by different authors, and the results regarding the relationship of SPX concentrations are still debated [14].

What seems to be equally important is the molecular role of SPX at both the central and peripheral tissue levels. Studies conducted so far have shown that SPX is expressed in neurons responsible for regulating the body’s homeostasis, which are located in the hypothalamus. SPX is attributed anorexigenic properties both by affecting NPY/AgRP, POMC, and orexin neurons [4,15,16], but also by affecting the modification of intracellular pathways in the liver, pancreas, adipose tissue, or muscles [11,12,17,18,19]. Interestingly, it has also been shown that the expression of SPX itself occurs in the supraoptic (SON) and paraventricular (PVN) nuclei of the human hypothalamus, which are crucial for the regulation of osmotic homeostasis, energy expenditure, nutritional behavior, reproduction, social behavior, and stress [20]. Considering the above, it should be emphasized that the role of SPX both at the molecular and peripheral levels seems to be crucial in the metabolism of the body; hence, there is a great need to expand knowledge about it.

In recent years, more and more attention has been paid to maternal and/or paternal metabolic imprinting, which may regulate and condition future metabolic changes in subsequent stages of a child’s life [21,22]. Despite the fact that spexin is a peptide described relatively recently, its role in the pathogenesis of obesity is significant. Therefore, we decided to check whether there is a correlation between the concentration of SPX in maternal blood serum and umbilical cord blood in obese and normal-weight mothers and whether these changes may correlate with the body weight of children in the future.

## 2. Materials and Methods

### 2.1. Study Participants and Ethics

The study was conducted on Caucasian women who were patients at the Gynecologic and Obstetrical University Hospital in Poznań due to planned deliveries. The research was conducted according to the principles stated in the Declaration of Helsinki. The protocol was approved by the Clinical Research Ethics Committee of Poznan University of Medical Sciences (approval number 997/18). The material for the study was maternal peripheral blood (MB) and umbilical cord blood (UBC) collected on the day of delivery from women of normal weight (BMI 18.5–24.9 kg/m^2^) and from obese women according to WHO standards (BMI value of ≥30 kg/m^2^) [23]. All pregnancies were carried to term and delivered naturally. Vaginal-assisted delivery and cesarean sections were excluded from the study. Exclusion criteria analogous to previous studies included in the Wojciechowska et al., 2022 manuscript were also used [24]. The exclusion criteria included, for example, twin pregnancy, operative delivery, premature birth, a child’s Abgar score lower than 9–10, diseases other than obesity (infections, preexisting chronic or gestational disease, type 2 diabetes), diseases other than obesity within 3 months of delivery, the use of drugs other than those supplements recommended during pregnancy, smoking during pregnancy, or alcohol consumption. Anthropometric parameters of mothers (age, height, body weight before pregnancy, BMI, and body weight on the day of delivery) and children (body mass, head, chest, abdominal, thigh, and arm circumference) were measured according to standard procedures. Moreover, after 36 months, the histories of the children’s body weight and height were collected. The data are presented in Table 1 and Table 2.

### 2.2. Umbilical Cord Blood (UCB) and Maternal Blood (MB) Collection

MB and UCB were collected using BD Vacutainer^®^ SST™ II Advance tubes (BD Diagnostics, Franklin Lakes, NJ, USA). The blood samples were left for 15–20 min at room temperature to form a blood clot and then centrifuged for 12 min at a speed of 3500× *g* at 4 °C. The serum samples were divided into portions, transferred to 0.5 mL tubes, and stored at −80 °C until analysis.

### 2.3. Metabolic and Hormonal Profile

Metabolic parameters were determined in the blood samples using commercially available colorimetric, Elisa, or RIA tests. The list of reagents used during the tests is given below: Poince Scientific (Lincoln Park, MI, USA)—glucose-oxy (cat. no.: G7521-100), cholesterol (cat. no.: C7510-100), triglycerides (cat. no.: T7532-100); Wako Pure Chemical Industries (Osaka, Japan)—NEFA; Sunred (Shanghai, China)—Human Spexin (cat. no.: 201-12-7257); Mediagnost (Reutlingen, Germany)—Adiponectin ELISA (cat. no.: E09); Merck Millipore (Burlington, MA, USA)—Multi-Species Leptin RIA (cat. no.: XL-85K) Human Insulin-Specific RIA (cat. no.: HI-14K). The optical density of samples for colorimetric and elisa assays was read on the Synergy 2 Microplate Reader (Biotek, Winooski, VT, USA). Gamma radiation from RIA samples was determined using the Wallac Wizard 1470 Gamma Counter (Perkin Elmer, Waltham, MA, USA).

### 2.4. Statistical Analysis

Statistical analyses were performed using the unpaired Student’s *t* test (two-tailed distribution) or the U Mann–Whitney test depending on the normality of the data distribution, and statistical significance was accepted at *p* < 0.05 (*) and *p* < 0.01 (**). Correlations between serum concentrations of SPX and other tested parameters were analyzed by Pearson’s correlation model and linear regression. All statistical analyses were performed using GraphPad Prism 6.0 software (GraphPad Software, San Diego, CA, USA).

## 3. Results

### 3.1. Metabolic and Anthropometric Parameters

The body mass and BMI measured before pregnancy were used as indicators of obesity in one group of analyzed women. An increased body weight in obese women compared to non-obese women was also observed on the day of birth (Table 1). There were no statistically significant differences between the antopometric parameters of newborns from different study groups (Table 2). Several parameters were evaluated to characterize the metabolic profile of the mother’s blood and umbilical cord blood, which are summarized in Table 3. There were statistically significant changes in cholesterol (*p* < 0.01) and triglycerides (*p* < 0.05) in the blood of non-obese and obese mothers.

### 3.2. SPX Concentration Changes

**Figure 1:**
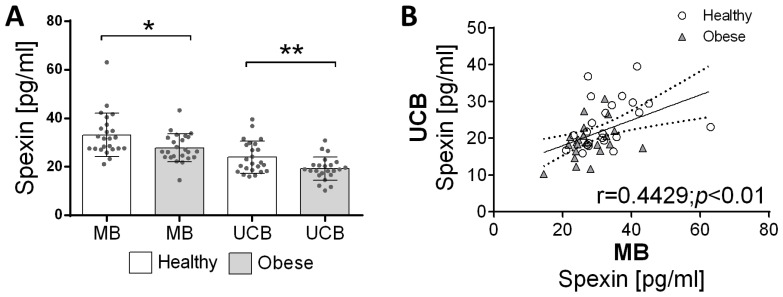
Changes in spexin concentration in MB and UCB in non-obese (Non-Ob) and obese (Ob) mothers (**A**). Values are presented as mean ± standard deviation of the mean. Statistically significant differences between the means for MB from Non-Ob and Ob subjects are marked * *p* < 0.05, and for UCB from Non-Ob and Ob subjects are marked ** *p* < 0.01. Correlations between spexin levels in the mother’s blood and umbilical cord blood (**B**). The r-value indicates a correlation, and the *p*-value indicates the significance of the correlation. MB—mother’s blood; UCB—umbilical cord blood.

Spexin concentrations were measured in MB and UCB from non-obese and obese subjects. A decrease in spexin concentration in maternal blood (*p* < 0.05) as well as in umbilical cord blood (*p* < 0.01) in obese subjects compared to non-obese subjects was demonstrated (Figure 1A). The correlation analysis of the spexin levels determined in all groups shows a positive correlation between the hormone levels in maternal blood and umbilical cord blood (r = 0.4429, *p* < 0.01, Figure 1B).

### 3.3. SPX and Body Weight

**Figure 2:**
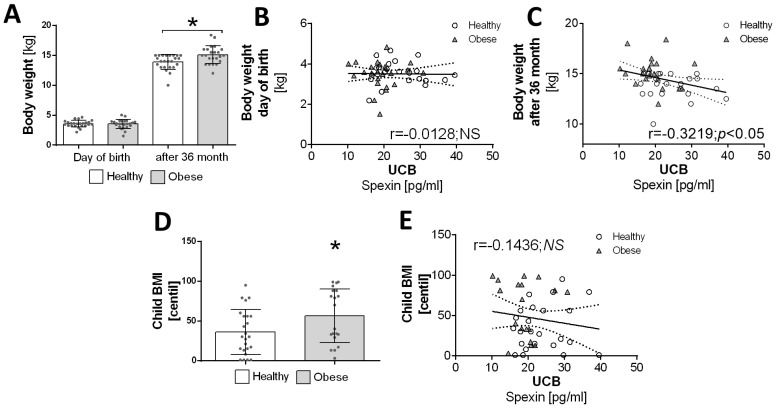
Changes in newborn body weight at birth and 36 months after birth in non-obese and obese mothers (**A**). Values are presented as mean ± standard deviation of the mean. Statistically significant differences between the means for newborn body weight from non-obese and obese subjects are marked * *p* < 0.05. Correlations between spexin in umbilical cord blood and newborn body weight (**B**) and between spexin and umbilical cord blood and body weight after 36 months after birth (**C**). Changes in child BMI in non-obese and obese subjects (**D**). Correlations between child BMI and spexin concentration in umbilical cord blood (**E**). The r-value indicates a correlation, and the *p*-value indicates the significance of the correlation. BMI—body mass index; MB—mother’s blood; UCB—umbilical cord blood. The r-value indicates a correlation, and the *p*-value indicates the significance of the correlation.

Next, we investigated the interactions between changes in body weight and spexin changes in maternal and umbilical cord blood. First, an increase in body weight at 36 months postpartum in obese subjects compared to non-obese subjects has been shown (*p* < 0.05, Figure 2A). Then, an analysis of the correlation between body weight and spexin concentration in umbilical cord blood shows no relationship in birth weight (Figure 2B); however, after 36 months after birth, a negative correlation was found (r= −0.3219; *p* < 0.05; Figure 2C). Then, BMI was measured in newborns, and an increase in this parameter was found in children from obese subjects (*p* < 0.05; Figure 2D). An analysis of the correlation of this parameter with the concentration of spexin in umbilical blood was also performed, but no statistically significant differences were found (Figure 2E).

### 3.4. Interaction with Leptin and Adiponectin

**Figure 3:**
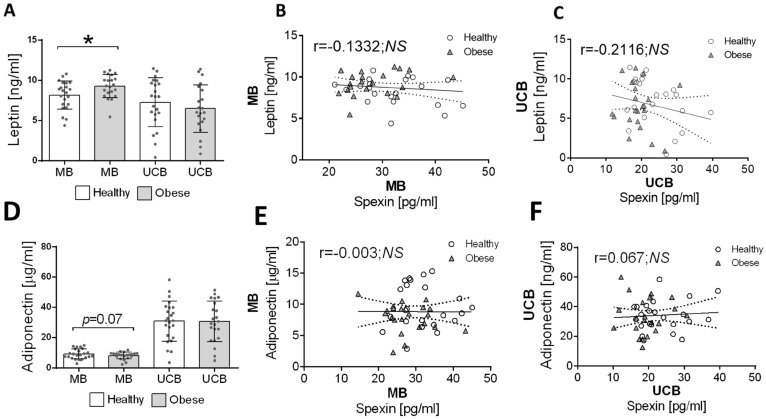
Changes in leptin (**A**) and adiponectin (**D**) concentrations in maternal and umbilical cord blood in non-obese and obese subjects. Values are presented as mean ± standard deviation of the mean. Statistically significant differences between the means for blood from non-obese and obese subjects are marked * *p* < 0.05. Correlations between leptin and spexin blood concentrations (**B**) or umbilical cord blood (**C**) concentrations for non-obese and obese subjects. Correlations between adiponectin and spexin blood concentration (**E**) or umbilical cord blood (**F**) concentration for non-obese and obese subjects.

Analysis of adipokine concentrations shows an increase in maternal blood leptin concentrations in obese subjects (*p* < 0.05; Figure 3A) but no changes in adiponectin concentrations (*p* = 0.07; Figure 3D). Subsequently, the analysis of the correlation between spexin and leptin shows no statistically significant differences in both maternal blood (Figure 3B) and umbilical cord blood (Figure 3C). A similar result was obtained by analyzing the correlation between adiponectin and spexin in maternal blood (Figure 3E) and cord blood (Figure 3F).

## 4. Discussion

For many years, there have been discussions about maternal and/or paternal factors that can condition/regulate the metabolism of children in the future. So far, a number of mechanisms have been discovered and described that affect the further development of the child and are conditioned by metabolic changes in the mother’s body, such as obesity, hyperglycemia, nutritional stress, and many others [25,26]. An increasing role is attributed to changes in the concentration of biologically active substances, such as hormones, peptides, and neropeptides, which can penetrate the placental barrier and affect both fetal development and the condition of the child’s future metabolism [27,28]. One of such peptides seems to be spexin, which is a strong stimulator of food intake as well as carbohydrate and lipid metabolism. Although the topic of SPX in the context of changes in its concentration in umbilical cord blood has already been discussed in the literature, we do not find data on obesity as a variable factor in these studies. The available data focus primarily on gestational age, preclampsia, type 2 diabetes, and metabolic syndrome as potential factors affecting this parameter [29,30,31,32].

This is particularly important due to the fact that disorders of the peptide and/or neuropeptide profile at the stage of fetal or infant life cause such disorders in older children, which are very often associated with metabolic disorders caused by increased body weight or obesity [33].

Using homogeneous groups of normal-weight and obese patients, we show for the first time that SPX concentrations are lower in serum blood as well as in umbilical cord blood obtained from obese mothers compared to non-obese women. Moreover, we also found a correlation between the UCB SPX level and the body mass of a child 36 months after birth.

Although SPX was discovered over 15 years ago, the manuscript of Walewski et al. from 2014 is considered a breakthrough in defining its function, in which they proved that the concentration of this peptide is reduced in obesity and that the gene encoding SPX is one of the most strongly downregulated genes in the tissue fat of obese people [1,2]. Our research also shows a decrease in the concentration of this peptide in the blood serum of obese women as well as in UBC. Although these changes were statistically significant, they oscillated around 15% and 20% in MB and UBC, respectively. This difference may not seem like a big difference. However, as indicated by the basic parameters after 36 months from birth, SPX may be a factor that determines the child’s metabolism in the future.

Already the first reports on SPX and its impact on the metabolism of obese people indicated its inverse correlation with leptin [2]. In addition, previous studies have shown that there may be a relationship between future obesity and the concentration of both leptin and adiponectin in umbilical cord blood, so we decided to check whether there is a similar relationship between the concentration of SPX and leptin or adiponectin in both maternal and umbilical cord blood [30,34,35]. Both our previous studies and other research groups in adult subjects show a strong or moderate inverse correlation between SPX and leptin, ranging from approximately −0.8 to −0.6. In contrast, our current study did not show a statistically significant relationship between SPX and leptin in both the blood of the umbilical cord as well as in the peripheral blood of healthy and obese mothers. The reason for the lack of correlation as well as significant changes between the groups of non-obese and obese women may be the date of collection (samples were taken on the day of delivery), where the mother’s body is exposed to many other factors that may affect the concentration of this peptide, such as stress factors or physiological changes of this peptide during pregnancy, where, as research has shown, the highest level of this adiponectin in blood serum occurs in the perinatal period [36,37,38,39].

It seems obvious that the role of SPX in newborns compared to children aged 3 years is slightly different. However, taking into account the observed correlations, the concentration of SPX during fetal life as well as in the first days of life may affect later development. To date, only a few functions of SPX in neonates have been described. So far, it has been found that SPX may be involved in the regulation of cell proliferation (e.g., in the adrenal cortex) and also affect postnatal hyperoxia-induced plasticity of the carotid body [40]. It was also shown that the concentration of SPX was lower in newborns with a small size compared to gestational age (SGA), in which the risk of obesity and overweight in the future is higher and correlated with length, body weight, and head circumference [30]. Another study that links changes in SPX concentration in newborns with oxygenation is the study by Gok et al., which showed that SPX concentration significantly changes in nondiabetic preeclamptic women, and the authors themselves link these changes with the hypoxic placenta [29]. Interestingly, using the H9C2 cell line and primary neonatal rat ventricular myocytes (NRVMs), it was shown that SPX can protect cardiomyocytes against the effects of hypoxia and mitochondrial dysfunction. Although this study was conducted in an in vitro system, taking into account the comparison of these results with the others, we can presume that the role of SPX in the fetus may be similar [41].

Although in our study we did not show changes in the weight of newborns from obese mothers and the presence of preeclampsia was an exclusion criterion during the study, we also observed significant changes in SPX concentration in newborns from obese mothers, which may suggest a very wide role of SPX in the metabolism of children both during the fetal and neonatal phases.

On the other hand, the role of SPX in the metabolism of a 3-year-old child may be slightly different, although there may also be potential similarities between the roles of SPX in fetal and infancy. One such similarity is the protective role of SPX on cardiomyocyte metabolism [42], since SPX, as mentioned above, can also play a role in infancy (development of heart cells). In addition, up to 36 months of age, adipose tissue develops, and the greater amount of brown adipose tissue that was present in the newborn is replaced by white adipose tissue. It seems so important that SPX is able to modulate both the metabolism of brown and white adipose tissue [12,19]. However, given the current limited knowledge of SPX, it is difficult to specifically identify differences in the role of this peptide at different stages of development. Therefore, it seems necessary to compare the results obtained with other potential indicators in the future, like molecular indicators including variants of different risk alleles to determine polygenic or common obesity, including *FTO*, *TNF-α*, *MC4R*, *ENPP1* genes, etc. [43,44]. Conducting more extensive research in the past also seemed to be a necessity.

We are also aware of the many weaknesses of our study. The first one that seems to be very significant is the size of the sample that was collected for the study. However, we treat these studies as preliminary studies that may be a clue for larger studies on the potential role of SPX during the development of fetuses in the mother’s body. In addition, another limitation of our research is the lack of determination of changes in this peptide during the entire pregnancy, but only on the day of delivery, where, as mentioned many times above, there are many other variables that can potentially affect the concentration of the tested peptides and other parameters, such as stress and many others. A very significant limitation of this study is the lack of other parameters apart from body weight and height, such as, e.g., SPX concentration, metabolic profile, and others, in children aged 36 months. In addition, we also do not have precise data on their diet or nutritional program, which could definitely enrich our results.

## 5. Conclusions

To conclude, pre-pregnancy obesity is associated with a significant decrease in maternal and umbilical cord blood spexin concentration, which is correlated with weight changes in children at 36 months of age, which may indicate that changes in the concentration of this peptide may be considered a potential marker of predisposition to obesity in the future. However, because these studies are preliminary and many inconsistencies remain to unfold, future studies are needed to elucidate the impact of maternal obesity on placental iron transfer and fetal iron metabolism.

## Figures and Tables

**Table 1:** Characteristic of mothers.

PARAMETER	Non Obese (n = 24)	Obese (n = 24)
**BEFORE PREGNECY**		
Women’s age (years)	30.21 ± 4.32	32.13 ± 5.08
Height (cm)	168.1 ± 5.06	167.0 ± 4.53
Body weight (kg)	64.41 ± 13.13	90.42 ± 12.96 **
BMI (kg/m^2^)	22.56 ± 0.86	32.37 ± 3.89 **
**ON THE DAY OF BIRTH**		
Body mass (kg)	78.42 ± 5.852	101.1 ± 11.42 **

Values are presented as mean ± SD. Statistically significant differences between MB from non-obese vs. obese patients are marked for *p* < 0.01 (**).

**Table 2:** Anthropometric parameters of newborns.

PARAMETER	Non Obese	Obese
Gender (M/F)	11/13	15/9
Head circumference (cm)	36.13 ± 2.11	36.33± 2.193
Chest circumference (cm)	34.08 ± 2.1	34.2 ± 2.345
Abdominal circumference (cm)	34.46 ± 3.027	35.22 ± 2.355
Thigh circumference (cm)	12.33 ± 1.027	12.49 ± 1.119
Arm circumference (cm)	10.13 ± 0.832	10.25 ± 0.925

Values are presented as mean ± SD.

**Table 3:** Metabolic profile of the patients.

Parameter	Non-Obese		Obese	
	MB	UCB	MB	UCB
Glucose (mg/dL)	97.04 ± 12.24	91.07 ± 11.57	103.3 ± 15.26	93.49 ± 17.62
NEFA (mmol/L)	0.7309 ± 0.33	0.4023 ± 0.15	0.7690 ± 0.38	0.4583 ± 0.21
Cholesterol (mg/dL)	177.1 ± 44.21	92.78 ± 32.51	236.7 ± 51.1 **	95.09 ± 18.02
Triglycerides (mg/dL)	221.3 ± 96.62	128.4 ± 73.93	307.5 ± 124.3 *	142.3 ± 106.1
Insulin (ng/mL)	13.09 ± 6.292	3.907 ± 3.390	13.79 ± 6.835	4.893 ± 4.051

Statistically significant differences between means for mother’s blood from NonOb and Ob subjects are marked where * *p* < 0.05 and ** *p* < 0.01. MB—mother’s blood; UCB—umbilical cord blood; NEFA—nonesterified fatty acids; NonOb—non-obese; Ob—obese.

## Data Availability

The row data presented in this manuscript are available on reasonable request from the corresponding author.
